# Microbiota and the Response to Vaccines Against Respiratory Virus

**DOI:** 10.3389/fimmu.2022.889945

**Published:** 2022-05-06

**Authors:** João I. B. Gonçalves, Thiago J. Borges, Ana Paula Duarte de Souza

**Affiliations:** ^1^Laboratory of Clinical and Experimental Immunology, Health and Life Science School - Pontifical Catholic University of Rio Grande do Sul (PUCRS), Porto Alegre, Brazil; ^2^Center for Transplantation Sciences, Department of Surgery, Massachusetts General Hospital, Harvard Medical School, Boston, MA, United States

**Keywords:** virus, microbiota, vaccine, respiratory virus, immune response

## Abstract

This mini review describes the role of gut and lung microbiota during respiratory viral infection and discusses the implication of the microbiota composition on the immune responses generated by the vaccines designed to protect against these pathogens. This is a growing field and recent evidence supports that the composition and function of the microbiota can modulate the immune response of vaccination against respiratory viruses such as influenza and SARS-CoV-2. Recent studies have highlighted that molecules derived from the microbiome can have systemic effects, acting in distant organs. These molecules are recognized by the immune cells from the host and can trigger or modulate different responses, interfering with vaccination protection. Modulating the microbiota composition has been suggested as an approach to achieving more efficient protective immune responses. Studies in humans have reported associations between a better vaccine response and specific bacterial taxa. These associations vary among different vaccine strategies and are likely to be context-dependent. The use of prebiotics and probiotics in conjunction with vaccination demonstrated that bacterial components could act as adjuvants. Future microbiota-based interventions may potentially improve and optimize the responses of respiratory virus vaccines.

## Introduction

Although it is a successful strategy, immune responses to vaccination can vary among individuals in the human population. One factor associated with this vaccine response variation is the microbiota composition. The human gut microbiota is composed of trillions of commensal microorganisms that can impact host physiology by metabolizing dietary components and synthesizing multiple metabolites that interact with host cells, including immune cells (1). The gut microbial community develops during early life together with the most extensive collection of immune cells in the body, the gut-associated lymphoid tissue (GALT), where they can influence the development of each other (2). However, the effects of the microbiome on the immune system go beyond acting locally in the intestine. Bacterial fragments and metabolites can translocate across the intestinal barrier, entering the circulation and reaching distant organs, including the lungs, where they can influence immune cells (3). Therefore, interventions targeting the gut microbiota may be one strategy to reduce disease burden and illness severity caused by respiratory tract infections (RTI) and increase vaccine responses against respiratory pathogens.

Most studies exploring the effects of the microbiota in immune responses focus on mucosal immunology in the gut, and responses to oral vaccination. However, the effect of the gut microbiota can go beyond intestinal tissues and influence responses to respiratory viruses’ vaccines. The lung microbiome is less diverse and contains smaller biomass than the gut (4, 5), but both present Bacteroidetes and Firmicutes as the dominant phyla (6–8). The colonization of the airways occurs shortly after birth, with the maturation of the microbiome occurring during the first 2-3 postnatal months in humans (9). The airway microbiota composition is mediated mainly by microbial immigration, microbial elimination, and the proliferation rate of bacteria (4, 5), with minimal contributions from selective pressures created by tissue growth conditions (7, 8). Thus, the gut and lung microbiota can have a role in the immune response during respiratory virus infection and the vaccine response against these pathogens. This mini review explores the current evidence relating microbiota composition, function, and its metabolites to the vaccine immune response against respiratory viruses, as well as the use of prebiotics and probiotics as natural adjuvants, describing the implication of this evidence on future research of vaccine development and potential interventions based on microbiota intervention.

## Scientific Background

### Microbiota, Respiratory Diseases and the Immune System

It is becoming more evident that changes in the gut microbiota composition are associated with respiratory diseases (10–12). Studies in mice using antibiotic treatment to deplete gut bacterial composition before airway infection challenge demonstrated that reducing intestinal microbiome diversity led to an increase in inflammation and mortality during the infection (13–15). An increase in *Bacteroidetes* and a decrease in *Firmicutes* phyla abundance in the gut was observed during respiratory syncytial virus (RSV) and influenza virus infections (16). In humans, alterations in alpha and beta diversity of gut microbial composition can also be observed during lung infections (17–19). In RSV infection, there was no significant difference in the abundance of microbes in the gut. However, the bacterial diversity had significantly changed, with an expansion of the families *Clostridiales*, *Odoribacteraceae*, *Lactobacillacea* and *Actinomyces* in infected patients compared to healthy controls (17). In COVID-19 patients, it was observed an increased abundance of *Streptococcus*, *Clostridium*, *Lactobacillus* and *Bifidobacterium* and a decrease of *Bacteroides*, *Roseburia*, *Faecalibacterium*, *Coprococcus* and *Parabacteroides* (18). Bacterial community richness and diversity were decreased in COVID-19 patients compared to healthy controls (19, 20). Certain intestinal commensal microorganisms associated with a healthy gut, such as *Faecalibacterium prausnitzii* and *Eubacterium rectale* and the butyrate-producing bacteria from the families *Lachnospiraceae* and *Ruminoccocaceae* were also decreased in COVID-19 and H1N1 patients (19, 21). Although the commonalities between the type of taxa that are changed during different diseases are present, most changes in microbial composition tend to be context-dependent and vary from one disease to another. The acknowledgment that multiple respiratory diseases present clinical intestinal manifestations (22, 23) indicates crosstalk at some level between these two mucosal surfaces. This crosstalk has been termed the gut-lung axis (3, 24–26).

The evolution of the immune system and the emergence of adaptive immunity coincided with the acquisition of a complex microbiota by the host (27), reinforcing the idea that the microbiome has a pivotal role in shaping the immune system. For example, leukocytes express a range of receptors that recognize microbial metabolites, conserved molecules associated with bacterial structural components (28) and other molecules synthesized by commensal bacteria. The disturbance of the normal microbiota, named dysbiosis, can dampen the host’s immune response, increasing susceptibility to complications during disease (29). Mice lacking microbiota (Germ-free [GF] mice) or antibiotic-treated mice have impaired responses to respiratory infections (30) and decreased IgA-producing plasma cells and the IgA repertoire (31, 32), confirming the idea that commensal bacteria can shape host immunity. Moreover, commensal bacteria regulate immune responses in the mucosal airways after influenza infection. Antibiotic treatment during respiratory infection in mice reduced the numbers of CD4+, CD8+ T cells, and IgA production. The number of dendritic cells (DCs) migrating from the lung to draining lymph nodes was also diminished. This effect was proposed to be mediated by a decreased production of inflammasome-related cytokines, such as IL-1b and IL-18, after antibiotic exposure (30). Interestingly, in the same study, it was reported that the administration of TLR agonists could rescue the immune responses against influenza infection after antibiotic treatment was also demonstrated by other groups (33).

The crosstalk between immune cells and commensal bacteria occurs mainly through cell adhesion contacts, especially in the mucosal sites, and through the interactions between microbial metabolites and receptors expressed by immune cells. One example of such metabolites is the short-chain fatty acids (SCFAs), a class of fatty acids produced by certain groups of bacteria after the metabolization of soluble and certain insoluble fibers ingested by the host (34). The SCFAs which have their physiologic role best described are acetic acid (acetate), butyric acid (butyrate), and propionic acid (propionate) (35). Through different mechanisms, these bacterial metabolites can influence different cellular processes, such as gene expression, proliferation, differentiation, and apoptosis (34, 35). One of these mechanisms is by acting on the cell surface, where the SCFAs can be sensed by G protein-coupled receptors (GPRs) expressed by several cell types, including immune cells. The GPR41 and GPR43 receptors can recognize all three SCFAs, whereas GPR109a can sense butyrate (35, 36). SCFAs can also enter cells *via* passive diffusion through the cell membrane or active transport by transmembrane proteins. Once inside, SCFAs can be metabolized by immune cells, increasing acetyl-CoA levels and subsequently fueling the TCA cycle and cell function (37). Intracellular SCFAs that are not metabolized can further migrate to the nuclei, acting as natural inhibitors of histone deacetylases (HDACs), leading to changes in gene expression patterns (36–38).

The SCFA butyrate was shown to suppress immune responses by promoting the expansion of regulatory T cells (Tregs) (39). Butyrate’s ability to induce Treg differentiation is in part mediated by its function as an HDAC-inhibitor, which in turn increases histone acetylation in the *Foxp3* and *Il10* gene loci, supporting the generation of Foxp3+ T cells (40, 41). Butyrate can also decrease the expression of inflammatory genes in neutrophils and macrophages, affecting their migration and cytokine production (42–44). In contrast, other studies reported that butyrate could increase macrophage antimicrobial response and promote T cell memory development (45, 46). Interestingly, CD8+ T cells failed to differentiate into memory cells during infection in mice lacking microbiota, and deletion of GPR41 and GPR43 also impaired these memory recall responses (46). Acetate was also shown to modulate memory T cell responses (47, 48), promote IgA production in a mechanism dependent on GPR43 expression in dendritic cells (DCs) (49), and protect against respiratory pathogens in a mouse model of bacterial infection (50). Mice treatment with the SCFA propionate could modulate hematopoiesis and increase the generation of dendritic cell precursors with an enhanced phagocytic activity that protects against allergic inflammation (51). A recent study from the same group demonstrated that treatment with a high-fiber diet, which is known to increase the systemic levels of SCFAs, also modulated hematopoiesis during influenza infection in mice, promoting a population of monocyte-derived macrophages that protected the legs from tissue damage caused by excessive neutrophil infiltration during infection. It also increased T cell cytotoxic activity (52). Aside from SCFAs, other microbial metabolites have also been reported to affect host immune responses, as reviewed elsewhere (53–55). For example, some metabolites can bind to the aryl hydrocarbon receptor (AHR) and are termed AHR ligands. These metabolites were reported to be involved in developing of immune responses (56). Activation of AHR is necessary for the generation of innate lymphoid cell (ILC) populations, particularly IL-22-producing group 3 ILCs (ILC3s) (57). Indoles are a class of metabolites that can modulate ILC3s and promote IL-22 secretion (58). In addition, indoles also enhance epithelial barrier function (59).

Taken together, these studies show that the presence of a healthy microbiota is essential for the normal development of the immune system and control of respiratory diseases, including protection against respiratory viral infections. The concept that microbial components can induce different immune responses opens the possibility of developing strategies to harness this immunomodulatory effect of the microbiome and use it in combination with other interventions that seek to produce an appropriate immune response, for example, during vaccination.

### Microbiota and Respiratory Virus Vaccine Responses

Vaccination is the most popular and cost-effective medical intervention, estimated to prevent more than 2 million deaths per year globally, and decrease disease severity (41, 60). However, the vaccinated individuals’ present variation in the immune response induced by vaccines. For example, antibody titers induced by yellow fever (61), seasonal trivalent inactivated influenza vaccine (TIV) (62) and hepatitis B (HepB) (63) vaccines were shown to vary more than 10-fold between individuals. The T cell response varied at similar levels to that of antibodies (64, 65). A better understanding of the mechanisms that lead to such variation is important to develop strategies that overcome these limitations.

The microbiome composition was associated with differences in responses to oral vaccination, including against respiratory pathogens. A seminal study in this field reported a correlation between the level of TLR5 expression and the antibody response against influenza hemagglutinin in humans after influenza vaccination (66). Subsequent work showing that TLR5-/- mice present an impaired capacity to mount antibody responses against TIV immunization confirmed the importance of this receptor for the generation of appropriate antibody responses after TIV immunization (67). The immune response was restored with the oral administration of flagellated, but not aflagellated strain of *E. coli*. Since flagellin is a well-known ligand for TLR5, this work indicates that PRRs derived from the gut microbiota can influence humoral responses to vaccinations against respiratory pathogens. Intriguingly, differences in intestinal microbiota composition did not affect responses against adjuvanted vaccines, demonstrating some limitations might exist in the extent to which commensal bacteria can modulate distal responses (67).

The use of antibiotics before or during vaccination was also demonstrated to modulate immune responses. In a randomized clinical trial, healthy individuals received a broad-spectrum antibiotic regimen (neomycin, vancomycin, and metronidazole) from three days before immunization until one day after (68). In this study, the lack of difference might be caused by pre-existing immunity to influenza, also known as immunological memory. The cells that mediate immune memory response were previously generated and present a more robust and rapid response during subsequent encounters with antigens. Thus, the authors suggested that this pre-defined immune memory may not be as susceptible as a primary response to the microbiota influence. To overcome this limitation, the authors conducted a second clinical trial only including individuals who had no exposure to influenza infection or vaccination in the preceding three years (68). These individuals had a marked decrease in the antibody titers before vaccination compared to the groups from the first study. The antibiotic-treated group had levels of IgG1 and IgA antibodies, and its avidity significantly decreased compared to the untreated group (68). These findings show that the impact of antibiotic exposure on the immune response during vaccination is limited by the presence of immunological memory, which seems to be more resilient to perturbations in the microbiota. In early life, when immunological memory is not well developed might be a better time for a microbiota intervention to increase vaccine response.

Since the emergence of COVID-19, a global effort has been made to try to understand how the disease develops and generate an effective vaccine. A portion of these studies focused on the associations between the vaccine response and the microbiota composition. One recent observational study reported that specific bacterial taxa were associated with the presence of higher neutralizing antibodies after vaccination. Interestingly, the microorganisms that positively correlated with neutralizing antibodies differed between the inactivated and the mRNA vaccines (69). This shows that the effects of different bacterial species in vaccine responses may be context-dependent. Also, the abundance of *Prevotella copri* and two species of *Megamonas* were reported to be increased in participants’ gut microbiota with fewer adverse effects to both vaccines (69). Further studies to understand the effects of gut commensal bacteria in COVID-19 vaccine responses are still ongoing. One ongoing observational study aims to analyze and compare the microbiome composition of subjects that received different COVID-19 vaccines and that recovered from the disease (70).

The findings discussed above show that the microbiota can modulate immune responses to vaccination. However, other factors known to influence these responses, such as route of administration, and genetic and environmental factors, may account for the variations observed. One limitation of these studies is that they are primarily cross-sectional, correlating the microbiome with vaccine responses at only one point in time. It is known that the microbiota composition can vary considerably over time depending on environmental exposure. For this reason, more longitudinal studies are needed to better evaluate the impact of the microbiota on vaccine responses.

The mechanisms associated with the effect of microbiota on vaccine response are not all described yet However, one explanation is that the microbiota constitutes a constant source of natural adjuvants that activate different pattern recognition receptors (PRRs) in host cells (71). One of these molecules, the muramyl dipeptide (MDP), present in the peptidoglycan of both Gram-positive and Gram-negative bacteria, is a ligand for the nucleotide-binding oligomerization domain containing 2 (Nod2) receptor and was implicated in enhancing antigen-specific IgG responses after intranasal immunization (72). Furthermore, Monophosphoryl lipid A (MPL), an agonist of TLR4 derived from *Salmonella enterica* endotoxin is already in clinical use as an adjuvant (73). Thus, alterations in the microbiota composition can change which PRRs and pathways are activated, leading to different innate and adaptive responses, potentially modulating vaccine responses.

The effect of microbiota on vaccine response and recent works showing how bacterial components can influence immunity led to increase in studies focusing on using prebiotic, probiotic and postbiotic treatments to increase vaccine responses (74). In the next section, we discuss some of the recent data regarding the effect of using these strategies in the context of vaccination against respiratory pathogens.

### Use of Probiotics and Prebiotics to Improve Vaccine Responses

A probiotic is any live organism that confers a health benefit to the host when administered in adequate amounts (75). Here, we will limit our discussion of probiotics as beneficial bacteria introduced into the host. In that sense, daily probiotic administration was shown to reduce the incidence of pathogenic bacteria in the nasal cavity (76). On the one hand, two meta-analyses analyzing various randomized clinical trials using probiotics reported their capacity to reduce respiratory infection duration and incidence (77, 78). In mice, the administration of probiotic strains of bacteria, such as *Lactobacillus*, as a prophylactic treatment was shown to increase innate and adaptive response against influenza during infection (79–81). On the other hand, studies with healthy individuals showed that probiotics were ineffective for reducing the incidence of respiratory tract infections (RTIs) (82–85). However, two of these studies reported that although not changing the incidence, probiotics reduced the duration and severity of respiratory infections (82, 83).

Probiotics in both oral and intramuscular vaccine formulations against HA and M2e influenza proteins boosted cellular and humoral responses in mouse and chicken models (86, 87). One study explored the feasibility of a new vaccine strategy using *Enterococcus faecium* as a bacterial vector carrying influenza antigens given orally and reported that this strategy induced antigen-specific antibodies and protected mice against H1N1 lethal infection (86). The addition of *Bacillus subtilis* spores into an intramuscular vaccine formulation of inactivated avian influenza virus led to enhancement of H9N2 virus-specific antibody IgG responses compared to the standard vaccine group (87). Further, another study using *Lactobacillus casei* demonstrated that bacterial microorganisms could be used as a vector to deliver antigens against respiratory pathogens either orally or intranasally administered and induce protective responses (88). These findings demonstrate that probiotic strains can be incorporated into vaccine components to help increase their efficacy.

To evaluate the use of probiotics to increase vaccine responses against respiratory pathogens, most studies relied on randomized clinical trials using intramuscular influenza trivalent inactivated vaccines (TIV) as their immunization model. Three independent studies using *L. paracasei* as their probiotic strain reported no differences in antibody and cellular responses to influenza after TIV vaccination (89–91). In contrast, two studies using a model of influenza vaccination administered intramuscularly found increased IgA antibodies in the saliva, higher seroconversion, and IgG titers when *L. paracasei* was administered orally with other probiotic strains (92, 93). Using *L. plantarum* as a probiotic strain, one study reported an increase in influenza-specific IgA and IgG antibodies six months after vaccination with a trivalent influenza vaccine (94). Other studies involving probiotics and responses to TIV and other vaccines to non-respiratory pathogens can be found in a meta-analysis (95) and systematic reviews (96, 97). An interesting observation from these studies is that the probiotic treatments generally confer beneficial effects to only one or two of the influenza strains present in the vaccine. Changes in the probiotic composition also modify the specificity of the increased response. More recently, an experimental study in mice showed that oral administration of the probiotic strain *Lactobacillus plantarum* GUANKE increased neutralization antibody levels and cellular immune response after intramuscular DNA vaccination against SARS-CoV-2 (81). Currently, there are several ongoing clinical trials assessing the utility of probiotics in enhancing antiviral immune responses to multiple SARS-CoV-2 vaccines (98). Thus, these results suggest that different probiotic species can be harnessed to increase specific memory responses to different strains of pathogens, although further work is needed to confirm this hypothesis.

Another strategy to expand certain commensal bacteria in the gut is prebiotics. These molecules are characterized as substrates, or nutrients, that can be metabolized by beneficial bacteria present in the host and therefore expand this specific microbial population (75). After birth, maternal milk can be considered a prebiotic, as it presents many components that help shape a healthy microbiota, such as oligosaccharides, IgA antibodies and hormones (99–101). Notably, these milk substrates were shown to induce the expansion of *Bifidobacterium* species of bacteria in the gut, which increased mucosal barrier function and protection against infections (102, 103). Breast milk also contains bacterial cells that initially colonize the infant’s gut and continue to influence gut microbiota composition throughout life (104). Examples of bacterial genera found to be supported by breast milk are *Veillonella* and *Rothia* (104), which are known to increase protection against asthma (105).

The use of prebiotics derived from fermentable carbohydrates and plant-based compounds was shown to reduce the incidence, duration, and severity of RTIs (95, 106–108). These studies show that this beneficial effect is mediated by alterations in the microbiota composition and suggests that changes in the host’s nutrition could also lead to similar results. Regarding the use of prebiotics in vaccination against respiratory pathogens, it was demonstrated that they could increase seroprotection against H1N1, but not the H3N2 and B strains of influenza after vaccination with TIV (97). No difference was observed in seroconversion rates. Furthermore, many dietary components other than prebiotics have immunomodulatory functions, including certain vitamins and carbohydrates (109, 110). These components should be investigated for their potential ability to increase vaccine responses. Thus, it will be essential to perform further studies to evaluate how the increase in the abundance of probiotic strains can increase immunity and if the use of bacterial metabolites can bypass the need to alter microbiome composition and still present beneficial effects.

## Conclusion and Perspectives

**Figure 1:**
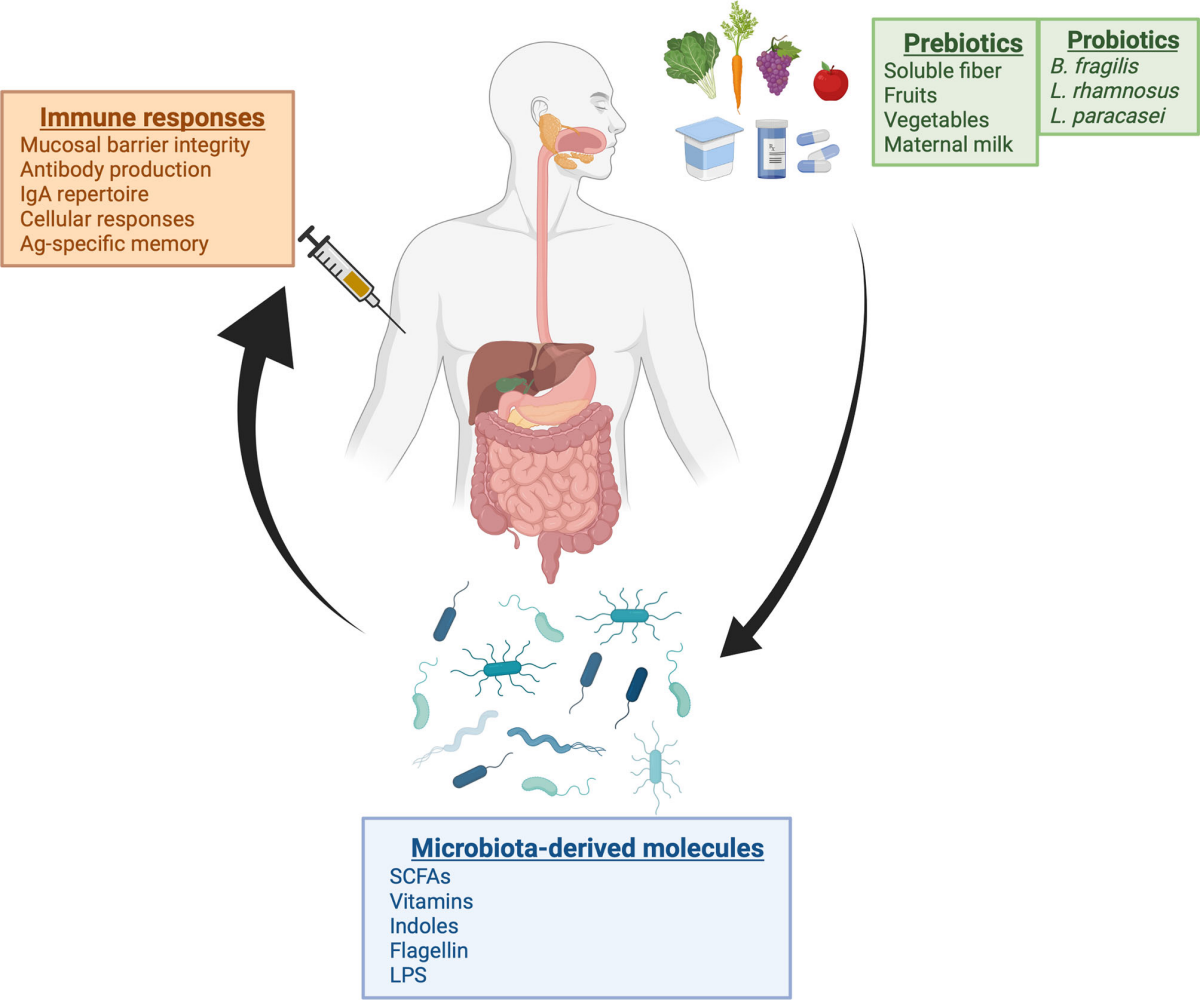
Schematic view of the microbiome-immunity relationship in the context of vaccination. Recent studies demonstrate that there is an association between microbiota and vaccine responses. Gut microbiome composition can be modulated through dietary intake and other environmental factors such as the use of antibiotics or probiotic consumption. With the discovery that abundance of specific bacterial taxa is correlated with increased immune responses after vaccination, therapeutic strategies using pre- or probiotics in combination with vaccination could represent an effective alternative to increase vaccine responses. Created with BioRender.com.

The microbiota composition is one of the factors that could contribute to some variability observed in vaccine responses. Recent studies demonstrated how microbiota could play a role in modulating the immune response elicited by vaccines. Those findings indicated that this effect is mediated by specific bacteria and depends on the context of the vaccine approach. Overall, these studies reinforce the idea that strategies to maintain a normal microbiota, including the use of pre- or probiotics, and the incorporation of bacterial molecules in the form of adjuvants into vaccine composition, can improve vaccine immune response (**Figure 1**). Since the beneficial effects of these bacterial metabolites come primarily from their interactions with cell surface receptors in host cells, the development and use of synthetic molecules that activate the same receptors with similar strength may be a feasible alternative to bypass the need to use bacterial cells to produce these components (111).

Despite the increasing knowledge that there is an association between a healthy gut microbiome and more effective responses to vaccination against respiratory pathogens, possible limitations of the applications of this as therapeutics should be noted. It is known that most probiotic microorganisms and bacterial metabolites applied in these studies have immunomodulatory effects, leading to a reduction in the overall inflammatory response. This suppression of inflammation could potentially lead to inadequate vaccine response. This idea is highlighted in the context of oral vaccines from studies reporting that oral vaccines perform poorly in developing countries (112–114), where the population is reported to have an increased gut microbiome diversity (115). The potential limitations of probiotics and their immunomodulatory effects in systemic vaccination modes should be further explored in the future.

Moreover, a decrease in inflammatory mediators does not necessarily preclude the development of an effective memory response. As we discussed in this mini review, bacterial metabolites such as SCFAs, which have regulatory effects, could also support the development of memory CD8^+^ T cells (46) and increase the production of antibodies by B cells (49). Immunomodulatory molecules, such as IL-10, are relevant for the maturation of the memory T lymphocytes population (116). Therefore, further work is needed to validate the applicability of these approaches and identify the limits of this microbiota-mediated modulation of immunity.

## Author Contributions

JG, TB, and AS wrote the paper. JG and TB did the figure. All authors contributed to the article and approved the submitted version.

## Funding

This study received funds from Coordenação de Aperfeiçoamento de Pessoal de Nível Superior (CAPES), finance code 1 and CNPq. TB effort was supported by NIH R01AI143887-04 (to LVR).

## Conflict of Interest

The authors declare that the research was conducted in the absence of any commercial or financial relationships that could be construed as a potential conflict of interest.

## Publisher’s Note

All claims expressed in this article are solely those of the authors and do not necessarily represent those of their affiliated organizations, or those of the publisher, the editors and the reviewers. Any product that may be evaluated in this article, or claim that may be made by its manufacturer, is not guaranteed or endorsed by the publisher.
